# Multifunctional flexible free-standing titanate nanobelt membranes as efficient sorbents for the removal of radioactive ^90^Sr^2+^ and ^137^Cs^+^ ions and oils

**DOI:** 10.1038/srep20920

**Published:** 2016-02-11

**Authors:** Tao Wen, Zhiwei Zhao, Congcong Shen, Jiaxing Li, Xiaoli Tan, Akif Zeb, Xiangke Wang, An-Wu Xu

**Affiliations:** 1 Institute of Plasma Physics, Chinese Academy of Sciences, Hefei, 230031, P.R. China; 2 Division of Nanomaterials and Chemistry, Hefei National Laboratory for Physical Sciences at Microscale, University of Science and Technology of China, Hefei, 230026, P.R. China; 3 NAAM Research Group, Faculty of Science, King Abdulaziz University, Jeddah, 21589, Saudi Arabia; 4 Collaborative Innovation Center of Radiation Medicine of Jiangsu Higher Education Institutions, P.R. China

## Abstract

For the increasing attention focused on saving endangered environments, there is a growing need for developing membrane materials able to perform complex functions such as removing radioactive pollutants and oil spills from water. A major challenge is the scalable fabrication of membranes with good mechanical and thermal stability, superior resistance to radiation, and excellent recyclability. In this study, we constructed a multifunctional flexible free-standing sodium titanate nanobelt (Na-TNB) membrane that was assembled as advanced radiation-tainted water treatment and oil uptake. We compared the adsorption behavior of ^137^Cs^+^ and ^90^Sr^2+^ on Na-TNB membranes under various environmental conditions. The maximum adsorption coefficient value (*K*_d_) for Sr^2+^ reaches 10^7^ mL g^−1^. The structural collapse of the exchange materials were confirmed by XRD, FTIR and XPS spectroscopy as well as Raman analysis. The adsorption mechanism of Na-TNB membrane is clarified by forming a stable solid with the radioactive cations permanently trapped inside. Besides, the engineered multilayer membrane is exceptionally capable in selectively and rapidly adsorbing oils up to 23 times the adsorbent weight when coated with a thin layer of hydrophobic molecules. This multifunctional membrane has exceptional potential as a suitable material for next generation water treatment and separation technologies.

Frequent oil spills and nuclear reactor accident have caused severe damage to the environment and ecosystems, and are difficult to clean up. Oil booms, skimmer vessels, combustion, and bioremediation are the most commonly used technologies oil spill remediation but often with poor efficiency^1^. In addition, the accident that occurred at the Fukushima Dai-ichi Nuclear Power Plant in Japan resulted in the release of artificial radioactivity to the environment. Consequently, the attenuation of long-lived radionuclides and various organic contaminants from aquatic environments has been an intensively pursued goal in water treatment^2^. Over the last decade, extensive studies have been devoted to the development of cost-effective alternatives. However, the limited sorption capacity and inconvenient sorption technologies have greatly hindered their practical application in the separation of radionuclides and oil.

Natural inorganic ion exchangers, such as zeolite-related materials, clay minerals, layered metal sulfide frameworks and titanate-based materials, have been intensively used for the uptake of radionuclides from nuclear wastewater. From the latter, a new class of materials, namely, titanate and its derivatives, has attracted tremendous attention because they are an extraordinarily effective scavenger for radionuclides and they have an outstanding affinity for heavy metal ions (Pb^2+^, Cu^2+^ and Hg^2+^)^3^,^4^. Emerging as new layered materials with exceptional ion exchange properties, titanates have the advantages of superior resistance to thermal exposure and radiation over the conventional organic ion-exchange resins^5^. The development of titanates with nanoscale dimensions and high morphological specificity, including one-dimensional (1D) titanate nanofibers (TNFs)^6^, nanotubes (TNTs)^7^ and nanosheets (TNSs)^8^ has been an effective strategy and could greatly contribute to the optimization of ion-exchange properties with greater selectivity for target cations and higher adsorption capacity because of their large surface area and unique TiO_6_ octahedral arrangement in layers yielding negative charges. Sodium ions or protons in titanate layers can be preferably exchanged with radioactive cations. However, the separation and recycling of the adsorbents remain a disadvantage, greatly restricting their practical applications.

The construction of a shape-moldable and nanoporous membrane is of great importance for applications to various fields in biological wastewater treatment^9^, gas separations^10^, catalyst supports^11^, and environmental remediation^12^. In this study, we fabricated flexible free-standing titanate nanobelt (TNB) membranes based on the layer-by-layer (LbL) assembly of TNBs and polyethylenimine (PEI). The multilayer TNB membrane can be cut into different diameter discs, which are further evaluated for the removal of radionuclides (^137^Cs^+^ and ^90^Sr^2+^) from simulated wastewater. Significantly, the TNB membrane exhibited rapid ion exchange kinetics and high adsorption capacities for both Cs^+^ and Sr^2+^ and can be easily separated from solution. In addition, a plausible ion exchange mechanism was proposed based on the X-ray diffraction (XRD) pattern, Raman spectroscopy and X-ray photoelectron spectroscopy (XPS) analyses. Moreover, the obtained TNB membrane can be used for large-area deposition of various materials. The membrane was coated with a thin layer of hydrophobic molecules (volatile silicone), resulting in a superhydrophobic surface, as evidenced by its high water contact angle. The modified TNB membrane was evaluated for the uptake of gasoline. This multifunctional membrane is expected to have potential as an excellent scavenger for cleaning up nuclear wastewater and oil spills.

## Results and Discussion

### Characterization of Na-TNB membrane

**Figure 1:**
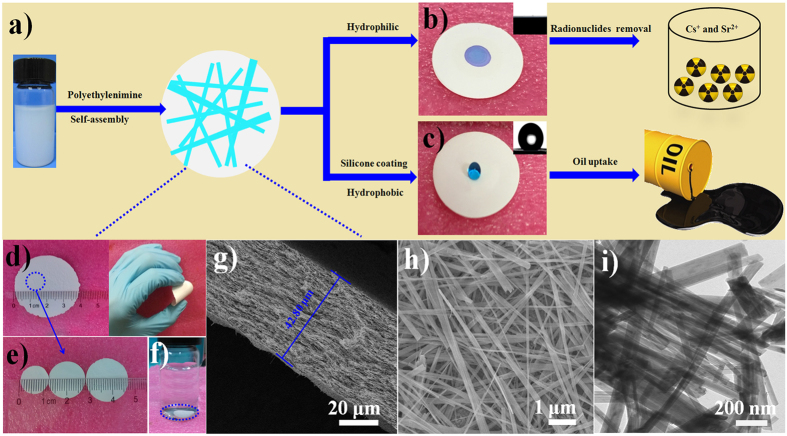
Characterization of sodium titanate nanobelt (Na-TNB) membrane. (**a**) The fabrication of Na-TNB membrane by vacuum filtration; photographs of the Na-TNBs membrane before (**b**) and after silicone coating (**c**) and their corresponding radionuclide removal and oil uptake studies, respectively; inset: water contact-angle measurement. (**d**) The flexible free-standing Na-TNBs membrane. (**e**) The Na-TNBs membrane with different diameter discs and (**f**) the stability of Na-TNBs membrane in solution. (**g**) The cross-sectional SEM image of Na-TNBs membrane and SEM (**h**) and TEM (**i**) images of Na-TNBs.

The layered Na-TNBs produced by the alkaline hydrothermal treatment were employed as the building blocks to construct a Na-TNB membrane. The Na-TNB membrane was obtained on a membrane filter through vacuum filtration based on the self-assembly of Na-TNBs and PEI in aqueous solution (Fig. 1a). The zeta potential of Na-TNBs dispersed in water is measured as −42.6 mV (Fig. S1). Benefiting from the highly negative surface charge of Na-TNBs, we can introduce the positive PEI molecules and stepwise construct multilayer assemblies of both of the components via electrostatic forces. The flexible free-standing Na-TNB membrane with a 4 cm diameter is shown in Fig. 1d. In addition, the diameters of the Na-TNB membrane can be tuned from 1.1 cm to 1.9 cm by cutting the fresh Na-TNB membrane into arbitrary sized discs using a precision disc cutter (Fig. 1e). The Na-TNB membrane shows a better mechanical property than the reported surface-sulfonated titanates and amine-tailored titanate nanotube membrane^13^,^14^ (Fig. S2). This is mainly attributed to the integration of PEI molecules into the Na-TNBs matrix. It can be seen that the Na-TNBs disc remains unaltered in aqueous solution for more than one week and can be further applied to remove ^137^Cs^+^ and ^90^Sr^2+^ from simulated wastewater (Fig. 1b,f). The cross-sectional FE-SEM image of Na-TNBs shows that the thickness of the membrane is approximately 42.8 μm (Fig. 1g). From the top view of the membrane (Fig. 1h), a large portion of the nanobelts agglomerate to form a network structure. Figure 1i shows a TEM image of Na-TNBs with diameters of 21–80 nm. The typical Brunauer−Emmet−Teller (BET) surface area of the as-prepared Na-TNBs is measured as 36 m^2^ g^−1^, showing type IV isotherms with type H3 hysteresis loops (Fig. S3a)^15^. The corresponding Barrett−Joyner−Halenda (BJH) analysis indicates that the Na-TNBs contain mesopores of ~10.7 nm in diameter (Fig. S3b). To obtain superhydrophobic surfaces, the hydrogen TNB membrane was functionalized with a hydrophobic PDMS coating through a traditional vapor deposition technique. The volatile silicone molecules were deposited on the hydrogen titanate nanobelts (H-TNBs) in a sealed Teflon-lined stainless-steel autoclave. The obtained H-TNBs through acid treatment retained the belt morphology (Fig. S4a and S4b). After silicone coating on H-TNBs, the surface of the H-TNBs became rough and the compact nanobelts were observed in the SEM image (Fig. S4c). As estimated by the TEM image (Fig. S4d), the vapour deposition process resulted in a conformal silicone layer coating on the surface of H-TNBs. To investigate the surface wettability, water droplets were brought into contact with the TNB membrane before and after the application of the silicone coating. As anticipated, it was observed that water droplets spread out completely, and the original Na-TNB membrane exhibited a low water contact angle (<1°) (inset of Fig. 1b). In contrast, the modified membrane became superhydrophobic^16^, and the water contact angle is approximately 150.58° in the inset of Fig. 1c. To evaluate their thermal stability, the obtained silicone coated H-TNBs and Na-TNBs were characterized by thermogravimetry-differential thermal analysis (TG-DTA) (Fig. S5). The result shows that the Na-TNB membrane is stable up to ca. 400 °C, whereas the methyl groups from the silicone backbone (Si-O-Si) are oxidized at higher temperature in the sample of silicone coated H-TNB membrane^17^. Due to their surface hydrophobicity, thermal and mechanical stability, the silicone-coated TNB membrane has potential applications for the efficient separation of organic liquids. In this study, the modified TNB membrane with a diameter of 1.9 cm showed an adsorption capacity up to 15 times its own weight for a variety of gasolines in 20 s. Therefore, the pristine Na-TNB membrane and the modified membrane can be used as adsorbents to remove various inorganic (heavy metal ions and radionuclides) and organic pollutants.

### Adsorption kinetics modeling studies

**Figure 2:**
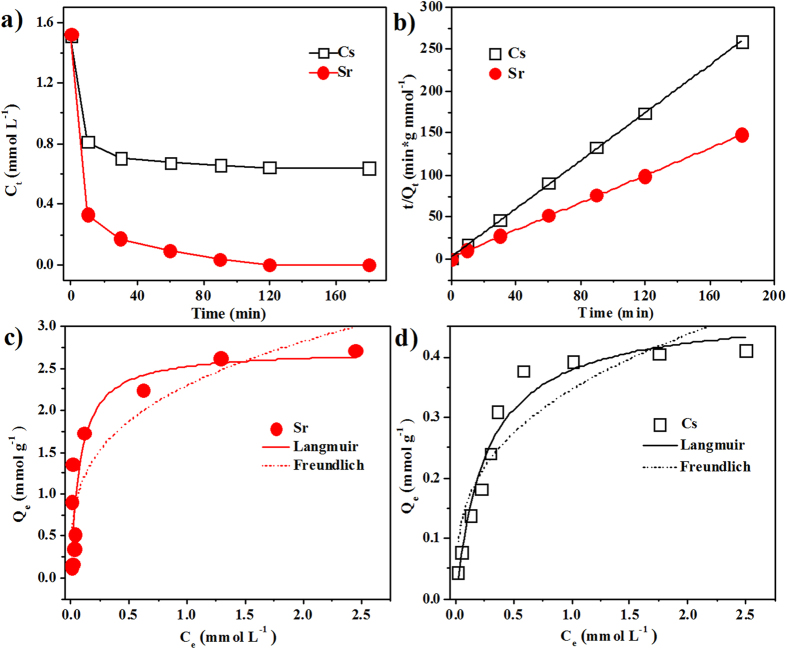
(**a**) Adsorption kinetic curves of Sr^2+^ and Cs^+^ onto Na-TNBs at 1.5 mmol L^−1^ initial Cs^+^ or Sr^2+^ concentration (*V*/*m* = 800 mL/g). (**b**) The linear fit of experimental data obtained using the pseudo-second-order kinetic model. Adsorption isotherms of Sr^2+^ (**c**) and Cs^+^ (**d**) onto Na-TNBs. Symbols denote experimental data, the solid lines represent the Langmuir model simulation, and the dashed lines represent the Freundlich model. All adsorption isotherms were conducted at *V*/*m* = 800 mL/g and with different initial Cs^+^ or Sr^2+^ concentration ranging from 0.17 mmol L^−1^ to 6.85 mmol L^−1^.

To evaluate the ion exchange properties of the Na-TNB membrane, ^137^Cs^+^ and ^90^Sr^2+^ were chosen as the target radionuclides to illustrate the adsorption performance. The adsorption kinetic curves of Cs^+^ and Sr^2+^ on the Na-TNB membrane were first investigated over 180 min (Fig. 2a). As shown in Table 1, the Na-TNB membrane was found to rapidly adsorb the radionuclides, and the amount of Sr^2+^ adsorbed on Na-TNBs reached 1.127 mmol g^−1^ within the contact time of 30 min at an initial concentration of 1.5 mmol L^−1^. More than 97.6% of Sr^2+^ was removed from the aqueous solution in 180 min of contact time. In comparison, the Cs^+^ adsorption reached equilibrium in 30 min, and the percentage of Cs^+^ was only 57.7% in this adsorption period. The different uptake efficiencies can be attributed to the affinity of the cations on the adsorbents, which can be expressed in terms of the distribution coefficient (*K*_d_)^18^. A material with a *K*_d_ value above 10^4^ mL g^−1^ is generally regarded as an excellent adsorbent^19^. For Sr^2+^ on Na-TNBs, the *K*_d_ value reached 4.5 × 10^6^ mL g^−1^, which is nearly three orders of magnitude higher than that of Cs^+^ (1.1 × 10^3^ mL g^−1^). This result is mainly ascribed to the higher affinity of Sr^2+^ than Cs^+^ for Na-TNBs. According to the stoichiometric nature of the ion exchanger, multivalent cations replace monovalent Na^+^ to decrease the number of ions in the interlayer, resulting in the shrinking of the basal spacing, which stabilizes the layer structure^3^. As a result, bivalent Sr^2+^ is preferred in the ion exchange over the monovalent Cs^+^, and higher valence ions can be effectively adsorbed on the Na-TNBs. The pseudo-second-order rate model can provide useful information for exploring the underlying mechanisms during the entire ion exchange process. Thus, the pseudo-second-order rate constants (*k*_2_) and the amounts of Cs^+^ and Sr^2+^ adsorbed at equilibrium (*Q*_e_) can be calculated using the intercept and slope of a plot of *t*/*Q*_t_ versus *t*^20^:


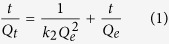


where *Q*_t_ and *Q*_e_ (mmol g^−1^) represent the sorption amount of target ions at time t (min) and the equilibrium time, respectively, and *k*_2_ (g mmol^−1^ min^−1^) is the kinetic rate constant. The kinetics of the ion exchange process is presented in Fig. 2b. The kinetic parameters obtained by fitting the experimental data are also listed in Table 1. The high correlation coefficient values (*R*_2_) reveal that the uptake of Cs^+^ and Sr^2+^ on Na-TNBs can be well simulated by a pseudo-second-order model^21^,^22^.

### Adsorption isotherm modeling studies

The quantities of Cs^+^ and Sr^2+^ associated with the Na-TNB membrane were determined from a supernatant analysis by varying the concentration of the target ions in solution. The corresponding Cs^+^ and Sr^2+^ adsorption isotherms are presented in Fig. 2c,d. Significantly, the adsorption of both of the target ions increased as the adsorbate concentrations increased. The adsorption data were simulated by the Langmuir and Freundich models to describe the adsorption data^23^:


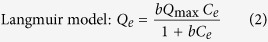



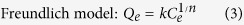


where *C*_e_ (mmol L^−1^) and *Q*_e_ (mmol g^−1^) are the radionuclide concentration at equilibrium and the amount of radionuclide adsorbed on the adsorbents per weight at equilibrium, respectively. *Q*_max_ (mmol g^−1^) is the saturation capacity at complete monolayer coverage, and *b* (L mmol^−1^) is a Langmuir constant related to the energy and affinity of the adsorbent. The Freundlich constant *k* is correlated to the relative adsorption capacity of the adsorbent (mmol g^−1^), and *n* represents the energetic heterogeneity. The corresponding parameters calculated from the model fitting are displayed in Table 2. It can be seen from the correlation coefficients (*R*^2^) that the Langmuir model fit the experimental data better than the Freundlich model. Thus, the Langmuir model can provide a good representation of the observed ion exchange process, which accounts for the homogeneous distribution of active sites on the Na-TNBs^24^. The adsorption capacity of Sr^2+^ determined by the Langmuir model was 2.72 mmol g^−1^, which is more than 5 times higher than that of Cs^+^ (0.48 mmol g^−1^). In addition, the Na-TNB membrane has *K*_d_ values >10^5^ mL g^−1^ for both of the radionuclides, suggesting that the obtained Na-TNBs are an exceptional adsorbent for the high concentrations of Cs^+^ and Sr^2+^ in aqueous solutions.

### Effect of adsorbent dosage and regeneration

**Figure 3:**
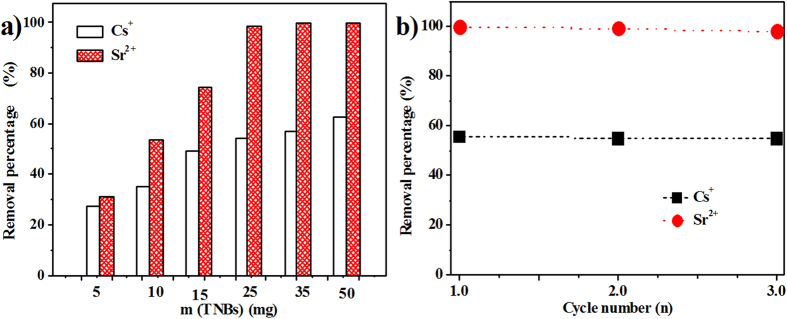
(**a**) The effect of adsorbent contents on the adsorption of Cs^+^ and Sr^2+^ at 1.5 mmol L^−1^ initial Cs^+^ or Sr^2+^ concentration and different *V*/*m* ranging from 400 mL/g to 4000 mL/g. (**b**) Recycling of Na-TNBs in the ion exchange of Cs^+^ and Sr^2+^ at 1.5 mmol L^−1^ initial Cs^+^ or Sr^2+^ concentration and *V*/*m* = 800 mL/g.

Figure 3a shows the effect of the contents on the adsorption of Cs^+^ and Sr^2+^ to explore the appropriate adsorbent dosage. As indicated in Fig. 3a, the percentage of radionuclide uptake as a function of the adsorbent content increases as the Na-TNB content increases. The phenomenon indicated that upon increasing the adsorbent amount, the number of surface active sites on the Na-TNBs increased, which was favorable for the binding of Cs^+^ and Sr^2+^. When the dosage of Na-TNBs is higher than 25 mg, more than 98.5% of Sr^2+^ is exchanged with Na^+^ in the interlayer. However, it was found that only 62.5% of Cs^+^ is removed at the maximum absorbent (50 mg). The same adsorbent dosage with a difference in ion exchange efficiency was observed, indicating a better retention for Sr^2+^ compared with Cs^+^. In general, the environmental sustainability and economic efficiency should be considered during the process of nuclear wastewater treatment. Thus, the regeneration and reusability of Na-TNBs were examined to evaluate its potential application. Sodium titanates can be easily regenerated by treating the Sr^2+^- and Cs^+^-laden product with a 1 M HCl solution and then performing a subsequent alkaline hydrothermal treatment^3^. Figure 3b shows the recycling of Na-TNBs in the removal of Sr^2+^ and Cs^+^ for three consecutive cycles. It can be seen that there is no obvious decrease in the removal of both of the cations, suggesting that the Na-TNBs possess exceptional regeneration ability for industrial applications.

### Adsorption mechanism

**Figure 4:**
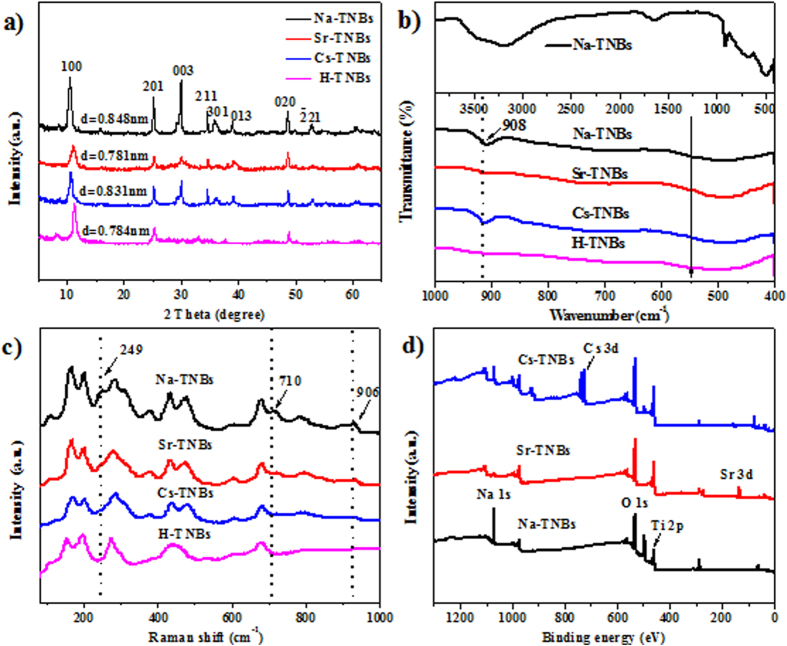
XRD patterns (**a**) FTIR spectra (**b**) and Raman spectra (**c**) before and after the ion exchange with Sr^2+^, Cs^+^ and H^+^, and XPS spectra (**d**) before and after the ion exchange with Sr^2+^ and Cs^+^.

The XRD patterns of the samples after radionuclide uptake showed significant changes. As can be seen in Fig. 4a, the peaks of Na-TNBs can be assigned to the typical primitive monoclinic Na_2_Ti_3_O_7_ phase (PDF Number: 72-0148)^6^. The interlayer distance of the (100) plane was estimated using Bragg’s law, and the corresponding *d*-spacing is 0.848 nm. The sodium ions and water molecules located between the Ti_3_O_7_^2−^ ions can be replaced by cations. Thus, it can be noted that the *d*_100_ spacing is much less than the Na-TNBs, and several diffraction peaks disappeared after uptake of the cations, which is mainly due to the collapse of the layered structure. The intense peak at 2*θ* = 28° is related to the sodium ions remaining in the interlayer space of the layered structure of titanate^25^. From the weak residual peak intensity, we can observe that the exchange of Sr^2+^ is more efficient than that of Cs^+^. The absence of a peak in the sample of H-TNBs can truly reflect the complete replacement of Na^+^ by H^+^ during acid treatment, which is in accordance with previous research^3^. Therefore, after the uptake of radionuclides, titanate can be treated by 1 M HCl and then used as a fresh Ti source to synthesize the titanate nanobelts.

The samples before and after ion exchange were also characterized by FTIR. The corresponding wide spectrum of Na-TNBs is shown in Fig. 4b. The absorption peak appearing at 908 cm^−1^ is ascribed to the stretching modes of the shortest Ti-O bonds, which is affected by the interlayer ions at the corners of the TiO_6_ slabs. The spectra analysis of samples between 1000 cm ^−1^ and 400 cm^−1^ are also given in Fig. 4b. The peak at 908 cm^−1^ after ion exchange becomes weaker, suggesting the partial replacement of Na^+^ by the target cations. This peak disappears in H-titanate due to the existence of a large amount of H^+^ in the interlayer, which is consistent with the result of the XRD analysis. The Raman spectra of Na-TNBs, H-TNBs, and after the adsorption of Cs^+^ and Sr^2+^ are presented in Fig. 4c. The Raman spectra reveal the typical peaks of sodium trititanate at 249 cm^−1^ (Na-O-Ti bond vibration) and 710 cm^−1^ (Ti-O-Ti stretching vibration in edge-shared TiO_6_ octahedra). The band at 906 cm^−1^ is generated by the symmetric stretching of short Ti−O bonds involving nonbridging oxygen atoms that are associated with Na^+^ 26. However, these peaks disappear after the exchange of target ions, suggesting the absence of a terminal oxygen atom in the corner-shared distorted TiO_6_ octahedron.

The XPS spectra further indicate the presence of a significant amount of Cs^+^ and Sr^2+^ in the used adsorbents (Fig. 4d). The strong high-resolution spectra of Cs 3d- and Sr 3d-anchored Na-TNBs are shown in Fig. S6a and S6b. The doublet peak characteristic of Sr 3d appears at 135.18 eV and 133.48 eV, assigned to 3d_3/2_ and 3d_5/2_, respectively. The Cs 3d_3/2_ (738.08 eV) and 3d_5/2_ (724.28 eV) peaks can also be observed in the spectrum of Cs-TNBs. The typical Na KLL peaks at 500 eV disappear after ion exchange with Cs^+^ and Sr^2+^, indicating that the sodium ions are nearly completely exchanged with the target cations^27^. From Fig. S6c and S6d, it can be clearly noticed that the high resolution O 1s and Ti 2p core level spectra are shifted to higher binding energies because of the interactions of radionuclides with the TiO_6_ octahedron. The sodium content decreases sharply, which demonstrates the exchange of Cs^+^ and Sr^2+^ with Na^+^ within the interlayer space. The deterioration of crystallinity and the decrease in diffraction intensity can also clarify the change of interlayer spacing from the above XRD patterns.

**Figure 5:**
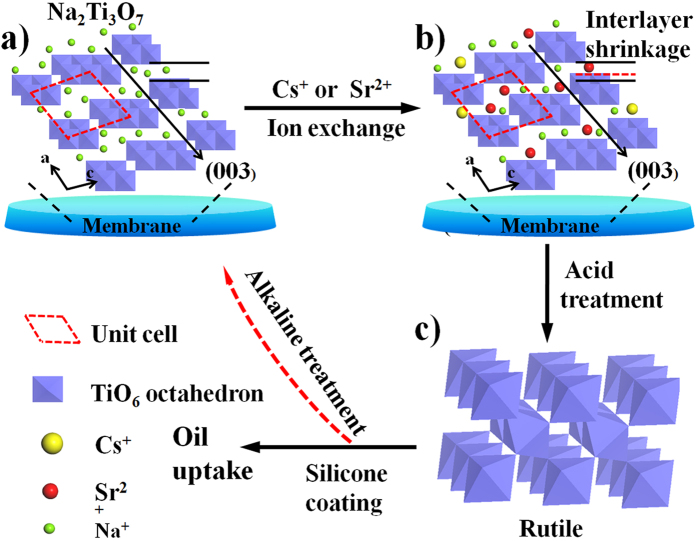
Schematic structural features of Na-TNBs before (**a**) and after ion exchange with Sr^2+^ and Cs^+^ ions (**b**). (**c**) The phase transition to the rutile in concentrate acid solution. And the rutile can be further used for the regeneration of the titanates through alkaline treatment and silicone coating for oil uptake.

The above results and discussion provide evidence of the ion exchange of Cs^+^ and Sr^2+^ on Na-TNBs based on batch adsorption and various characterization methods. According to previous studies, the structures of titanate before and after ion exchange of radioactive ions are presented in Figure 5^28–30^. The exchange of Cs^+^ and Sr^2+^ ions occurs within the (003) planes of titanate nanobelts. As noticed from the XRD patterns (Fig. 4a), the decrease in the (003) diffraction intensity caused by Sr^2+^ exchange product is much lower than that caused by Cs^+^ exchange^31^. Because the diameter of the Na^+^ ion is 1.02 Å, the Cs^+^ ion (with a diameter of ~3.38 Å) will be more difficult to exchange than the Sr^2+^ ion (with a diameter of ~2.26 Å)^32^. The replacement of Na^+^ in the interlayer resulted in the interlayer shrinking, while the decrease of basal spacing could stabilize the layer structure (Figure 5). In general, the presence of hard cations, such as Na^+^, Mg^2+^ and Ca^2+^, in polluted wastewater at high levels causes the traditional adsorption technology to be inefficient^33^. The absolute hardness of Sr^2+^ is 16.3, which is much softer than that of Na^+^ (21.1). Therefore, the cations with higher valence, smaller radius and lower hardness are preferred in the ion exchange process. As the smallest cation, H^+^ is generally used to regenerate adsorbent and to desorb the adsorbate from materials, resulting in the highest priority in the ion exchange^3^. Meanwhile, the titanate phase transition to the rutile phase occurs through the concentrated acid treatment (Figure 5). The interlayer distance of H-TNBs (0.784 nm) further supports the formation of H_2_Ti_3_O_7_^34^. The rutile phase can be used either as the precursor to coat silicone for the oil uptake studies or as the new Ti source to regenerate the Na-TNBs through alkaline treatment for the next cycle.

### Oil uptake with silicone coated TNB membrane

**Figure 6:**
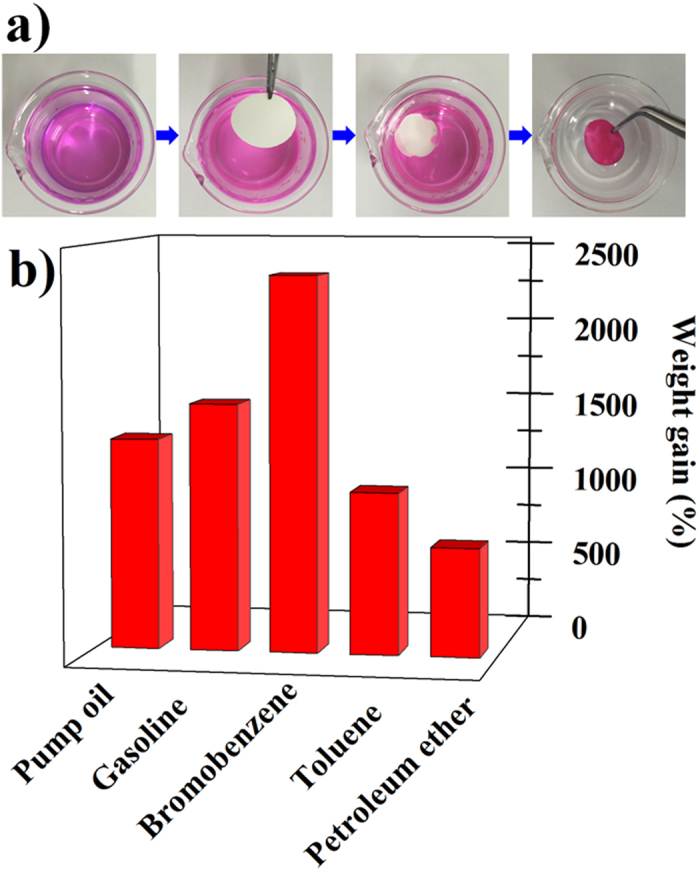
(**a**) A layer of gasoline was absorbed by the modified TNB membrane in 15 s. The gasoline was labeled with Rhodamine B for clear presentation. (**b**) Absorption efficiency of modified TNB membrane for oils and organic liquids.

The adsorption efficiency, referred to as weight gain, wt (%), was defined as the weight of absorbed oils or organic liquids per unit weight of modified TNB membrane. When brought into contact with a layer of gasoline (dyed with Rhodamine B) on a water surface, the modified TNB disk completely absorbed the gasoline in 20 s, yielding clean water that was originally contaminated by the gasoline (Fig. 6a). Compared with the infiltration of the Na-TNB membrane in water (Fig. 1f), it can be significantly observed that the disk floats on the water surface due to its low density and hydrophobic properties. To evaluate the adsorption capacities, various classes of oils and organic liquids were investigated. As shown in Fig. 6b, the disk can absorb the liquids in amounts up to 6.8–23 times its weight for a variety of organic solvents and oil. Specifically, the uptake capacities of pump oil and gasoline oil are 13 and 15 times its own weight, respectively. In addition, the TNB disk exhibits a significantly higher uptake capacity of bromobenzene (23 times) than that of toluene (10 times) and petroleum ether (6.8 times). These membranes could be easily separated from aqueous solution using tweezers, which provides an immediate application for the removal of hydrophobic contaminants from water. Therefore, the silicone-coated TNB membrane may be a promising candidate for the highly efficient separation/extraction of specific substances.

In conclusion, the hydrothermal preparation of layered Na-TNBs in a relatively weak alkaline solution was conducted. Subsequently, the assemblies of Na-TNBs and PEI were used to construct the flexible free-standing Na-TNB membrane. These membranes can also be coated with a thin layer of volatile silicone, resulting in a superhydrophobic surface with a high water contact angle (150.58°). The absorption capability of the modified membrane can reach 6.8–23 times its own weight for oils and organic liquids. The ion exchange behavior of Na-TNBs towards radioactive Cs^+^ and Sr^2+^ and the modified membrane towards gasoline were evaluated at room temperature, respectively. The results show that the Langmuir adsorption isotherm could describe the ion exchange processes, and the kinetics of ion exchange followed the pseudo-second-order rate model. A maximum adsorption capacity of 2.72 mmol g^−1^ was achieved on Na-TNBs toward Sr^2+^, which was nearly 5 times higher than that of Cs^+^. As a result, the radius, hardness, and valence of the cations are the primary factors affecting the ion exchange process. The multifunctional membrane could be a suitable material for radionuclide environmental pollution purification and for application to the removal of organics, particularly in the cases of oil spill cleanup.

## Methods and Materials

### Fabrication of a multilayer TNB membrane

The pristine sodium TNBs were synthesized through an alkaline hydrothermal treatment^3^,^35^. To obtain long sodium titanate nanobelts (Na-TNBs), 1 g of P25 powder (Degussa AG, Germany) was dispersed in 80 mL of a 5 M NaOH solution under vigorous stirring for 30 min and hydrothermally treated at 200 °C for 72 h in a Teflon-lined autoclave. After the reaction, the white precipitate was recovered by filtration and was washed with deionized water until reaching pH ~12; it was then rinsed with ethanol to remove the residual surface (OH^−^ and Na^+^) and freeze-dried.

To prepare the cross-linked TNB membrane, 40 mg of TNBs was dispersed in 40 mL of deionized water under ultrasonication for 1 h to form a suspension. To the above solution was added 1 mL of a polyethylenimine (PEI, MW 1800) solution (2.5 g L^−1^), which was then stirred for 1 h at room temperature. In a typical preparation process, the above suspension was vacuum filtered through a membrane with an average pore size of 0.22 μm (Millipore). The cross-linked films were further dried at room temperature and peeled from the membrane.

### Silicone coating process

The TNB membrane coated with hydrophobic silicone was obtained using a traditional vapor deposition technique^36^. The Na-TNBs were first dispersed in 1 M HCl to yield hydrogen TNBs through ion exchange of Na^+^ with H^+^ in the titanate tunnels of the nanobelts. The layered protonated TNB membrane was obtained by the aforementioned procedure and was then transferred along with polydimethysiloxane (PDMS) to a sealed Teflon-lined stainless-steel autoclave. The autoclave was heated to 234 °C and was maintained for 1 h. During this time, a mixture of low molecular weight and volatile silicone from the thermal degradation of PDMS formed a conformal layer on the surface of the TNB membrane.

### Characterization

Field emission scanning electron microscopy (FE-SEM) was performed with a JEOL JSM-6330F system at a beam energy of 15.0 kV to characterize the morphologies and sizes of the Na-TNBs. Transmission electron microscopy (TEM) (JEOL-2010) was used to observe the microstructures by drying a droplet of the Na-TNB suspension on formvar-coated copper grids. Thermogravimetric analysis (TGA) was performed on TGA Q5000IR (TA Company, USA) under air flow with a temperature rising rate of 10 °C min^−1^. X-ray diffraction (XRD) patterns were collected with a Philips X’Pert Pro Super X-ray diffractometer with Cu-Kα radiation between 5° and 65° at a scan rate of 0.02° s^−1^. The Fourier transform infrared (FTIR) spectrum (400 to 4000 cm^−1^) was measured using a Nicolet Magana-IR 750 spectrometer with pure KBr as the background. Raman spectra were obtained with an InVia microscopic confocal Raman spectrometer (Renishaw, England) using green 514.5 nm laser excitation. The surface states of the samples were characterized by X-ray photoelectron spectroscopy (XPS) using a VG Scientific ESCALAB Mark II spectrometer equipped with two ultrahigh vacuum (UHV) chambers. Contact angles were measured with a contact angle meter SL200B (USA KINO INDUSTRY Co., Ltd.). The water droplet volume was fixed at 3.0 μL, and the contact angle was determined 3 s after the attachment to the membrane surface. Static mechanical uniaxial in-plane tensile test was conducted with a dynamic mechanical analyzer (DMA Q800, TA Company, USA) with a preload of 0.01 N at a loading rate of 0.5 N min^−1^. The zeta potential was measured using a ZETASIZER 3000 HSA system.

### Batch adsorption experiments

The uptake of radionuclides at various concentrations (0.17–6.85 mmol L^−1^) from aqueous solutions was performed using a batch technique at room temperature and *V*/*m* = 800 mL g^−1^. The Na-TNB weight was maintained at 25 mg. After immersing the membrane in the simulated wastewater for 24 h, the supernatant was retrieved, and the amount of residual ^137^Cs^+^ or ^90^Sr^2+^ was determined by liquid scintillation counting using a Packard 3100 TR/AB Liquid Scintillation analyzer. The removal percentage was calculated from the difference between the initial concentration (*C*_0_) and the equilibrium concentration (*C*_e_):


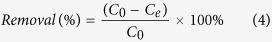


The results of the kinetic studies suggested that the adsorption of ^137^Cs^+^ or ^90^Sr^2+^ on Na-TNBs achieved equilibrium in several hours. For each experiment, 25 mg of Na-TNBs was immersed in a 20 mL solution containing adsorbate (1.5 mmol L^−1^) with different contact times (15–180 min). To investigate the effect of adsorbent content, the Na-TNB membrane was cut into discs with different masses (5–50 mg). The distribution coefficient (*K*_d_) was applied to understand the affinity of the Na-TNB membrane for ^137^Cs^+^ or ^90^Sr^2+^ adsorption:


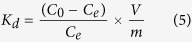


For the regeneration experiments, the Na-TNBs after the uptake of radionuclides were treated with 1 M HCl. The protonated titanates can be used as new Ti precursors to synthesize Na-TNBs. H-titanate nanobelts were modified by the aforementioned silicone coating procedure for organic liquid and oil uptake studies. The gasoline for this study was purchased from a China Sinopec gas station.

## Additional Information

**How to cite this article**: Wen, T. *et al.* Multifunctional flexible free-standing titanate nanobelt membranes as efficient sorbents for the removal of radioactive ^90^Sr^2+^ and ^137^Cs^+^ ions and oils. *Sci. Rep.* **6**, 20920; doi: 10.1038/srep20920 (2016).

## Supplementary Material

Supplementary Information

## Figures and Tables

**Table 1:** Kinetic data of Cs^+^ and Sr^2+^ adsorption on Na-TNBs: the distribution coefficient,*K*_d_, pseudo-second-order rate constants, *k*_2_, and correlation coefficient values,*R*^2^.

*C*_0_ (mmol L^−1^)1.5	*Q*_t_ (mmol g^−1^)	Removal (%)	*K*_d_ (mL g^−1^)	*k*_2_ (g mmol^−1^ min^−1^)	*R*^2^
Cs^+^	0.695	57.7	1.1 × 10^3^	0.6988	0.9996
Sr^2+^	1.127	97.6	4.5 × 10^6^	0.2964	0.9992

**Table 2:** The parameters calculated from the Langmuir and Freundlich isotherm models for the Cs^+^ and Sr^2+^ adsorption on Na-TNB membrane.

Species	Langmuir			Freundlich		
	*Q*_max_ (mmol g^−1^)	*b* (L mmol^−1^)	*R*^2^	*k*	*n*	*R*^2^
Cs^+^	0.48	3.76	0.992	0.35	2.98	0.843
Sr^2+^	2.72	12.68	0.994	2.30	3.37	0.881
