# Supplementary material for: Multifunctional flexible free-standing titanate nanobelt membranes as efficient sorbents for the removal of radioactive 90Sr2+ and 137Cs+ ions and oils
Source: Sci Rep. 2016 Feb 11;6:20920. doi: 10.1038/srep20920 (PMC4750037; doi:10.1038/srep20920)
Supplement: Supplementary Information [file srep20920-s1.doc]

**Supporting Information on**

**Multifunctional fle****xible free-standing titanate nanobelt membranes as efficient sorbents for the removal of radioactive ^90^Sr^2+^ and ^137^Cs^+^ ions and oils**

**Tao Wen^1^, Zhiwei Zhao^2^, Congcong Shen^2^, Jiaxing Li^1^, Xiaoli Tan^1,4^,*, Akif Zeb^2^, Xiangke Wang^1,3,4^,* & An-Wu Xu^2^,***

^1^Institute of Plasma Physics, Chinese Academy of Sciences, Hefei, 230031, P.R. China.

^2^Division of Nanomaterials and Chemistry, Hefei National Laboratory for Physical Sciences at Microscale, University of Science and Technology of China, Hefei, 230026, China.

^3^NAAM Research Group, Faculty of Science, King Abdulaziz University, Jeddah, 21589, Saudi Arabia.

^4^Collaborative Innovation Center of Radiation Medicine of Jiangsu Higher Education Institutions, P.R. China

Correspondence and requests for materials should be addressed to X.T. (tanxl[@ipp.ac.cn](mailto:xkwang@ipp.ac.cn)) or X.W. (xkwang@ipp.ac.cn) or A.-W.X. ([anwuxu@ustc.edu.cn](../anwuxu@ustc.edu.cn))


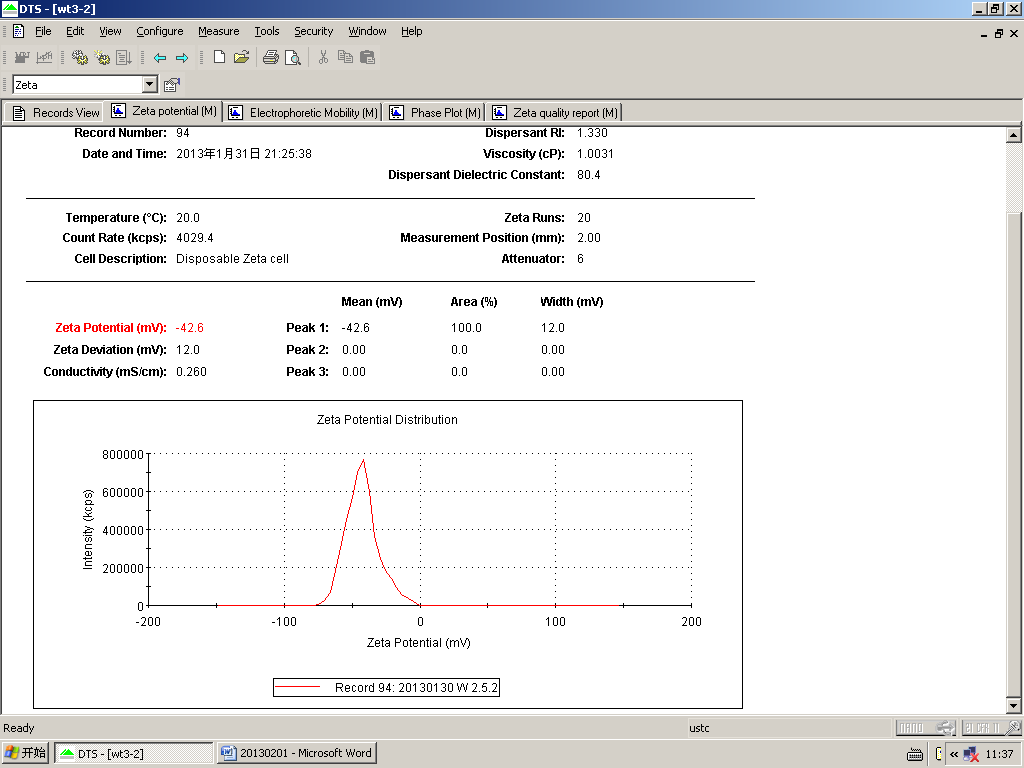


**Figure S1.** Zeta potential of Na-TNBs dispersed in water.

**Figure S2.** Stress–strain curves of the Na-TNBs membrane.

From the stress–strain curve of Na-TNBs membrane, the fracture strength of membrane was 17.89 MPa, higher than the previously reported values of surface-sulfonated titanates and amine-tailored titanate nanotube membrane^1,2^. The better mechanical properties is mainly attributed to the integration of PEI molecules into the Na-TNB matrix.

**Figure S3.** Nitrogen adsorption/desorption isotherms of Na-TNBs (a) and pore distribution in Na-TNBs (b).


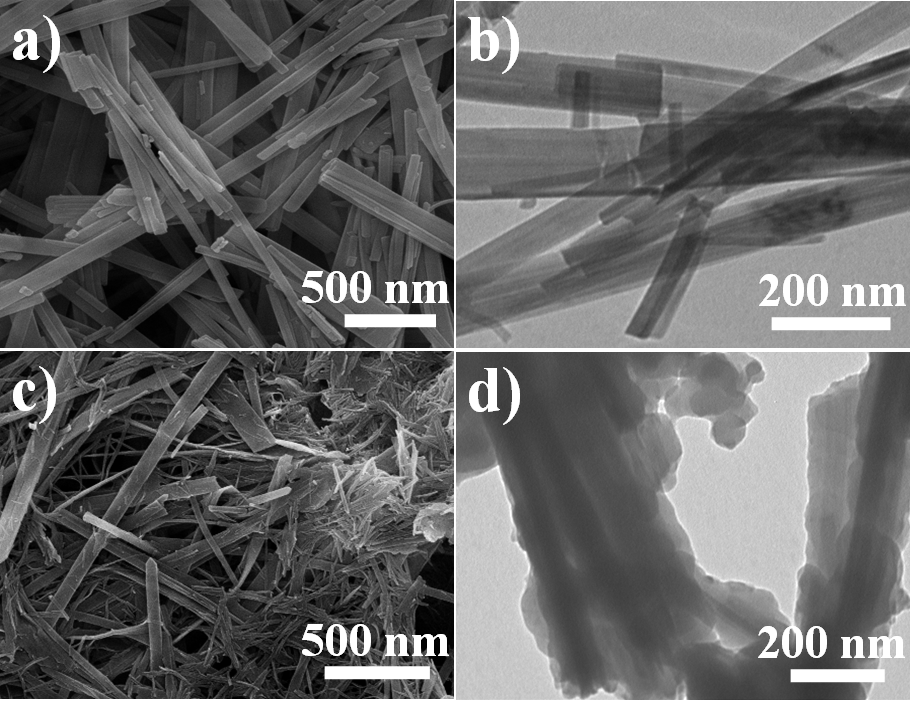


**Figure S4.** SEM (a) and TEM (b) images of H-TNBs, and SEM (c) and TEM (d) images of silicone coated H-TNBs.

**Figure S5.** TG/DTA curves of the Na-TNBs (a) and silicone coated H-TNBs (b).

To evaluate their thermal stability, the obtained Na-TNBs and silicone coated H-TNBs were characterized by thermogravimetry-differential thermal analysis (TG-DTA) under air flow with a temperature rising rate of 10 ^o^C min^-1^. As shown in Fig. S5, the curves reveal that the loss of physically and chemically adsorbed water below 400 ^o^C. The result shows that the Na-TNBs membrane is stable up to ca. 400 ^o^C, whereas the methyl groups from the silicone backbone (Si-O-Si) are oxidized at higher temperature in the sample of silicone coated H-TNBs membrane^3^.


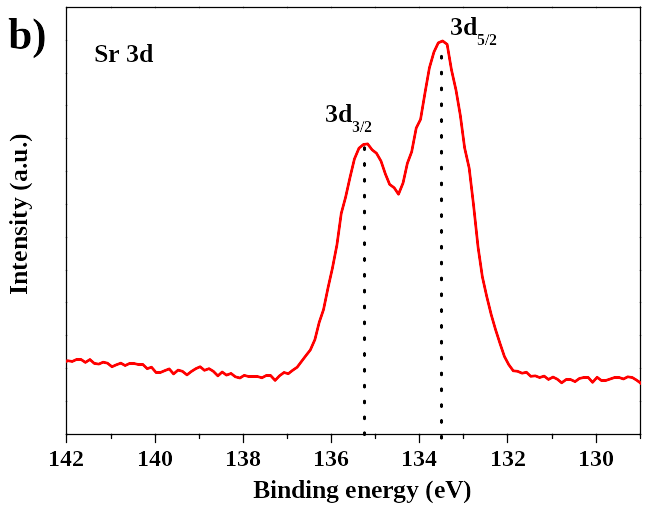

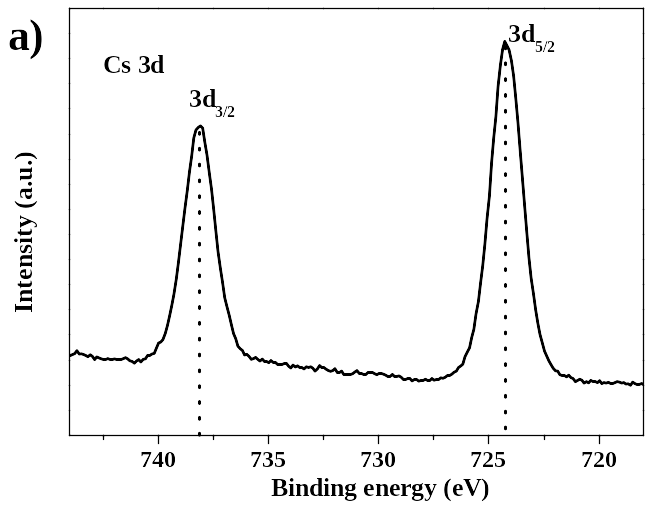

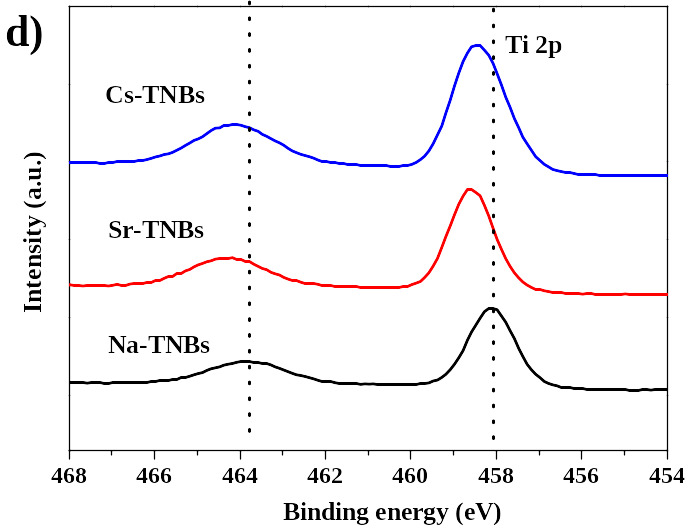

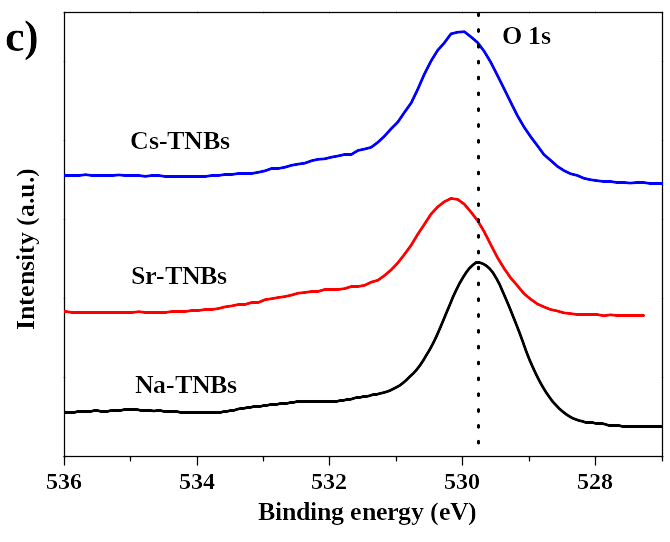


**Figure S6.** XPS high-resolution spectra (a) Cs 3d and (b) Sr 3d after the ion exchange. (c) O 1s, and (d) Ti 2p before and after ion exchange.

**References**

1. Rhee, C. H., Kim, Y., Lee, J. S., Kim, H. K. & Chang, H. Nanocomposite membranes of surface-sulfonated titanate and Nafion for direct methanol fuel cells. *J. Power Sources* **159**, 1015–1024(2006).

2. Li, Q. et al. Impregnation of amine-tailored titanate nanotubes in polymer electrolyte membranes. *J. Membrane Sci.* **423**, 284-292(2012).

3. El-Hag A. H., Simon, L. C., Jayaram, S. H., & Cherney, E. A. Erosion resistance of nano-filled silicone rubber. *IEEE Transactions on Dielectrics and Electrical Insulation* **13**, 122–128(2006).
